# Characterization of airag collected in Ulaanbaatar, Mongolia with emphasis on isolated lactic acid bacteria

**DOI:** 10.1186/s40781-016-0090-8

**Published:** 2016-03-08

**Authors:** Suk-Ho Choi

**Affiliations:** Animal Science and Biotechnology, Sangji University, Wonju, 26339 South Korea

**Keywords:** Airag, Koumiss, Lactic acid bacteria, Lactose, Acid production

## Abstract

**Background:**

Airag, alcoholic sour-tasting beverage, has been traditionally prepared by Mongolian nomads who naturally ferment fresh mares’ milk. Biochemical and microbiological compositions of airag samples collected in Ulaanbaatar, Mongolia and physiological characteristics of isolated lactic acid bacteria were investigated.

**Methods:**

Protein composition and biochemical composition were determined using sodium dodecyl sulfate-gel electrophoresis and high performance liquid chromatography, respectively. Lactic acid bacteria were identified based on nucleotide sequence of 16S rRNA gene. Carbohydrate fermentation, acid survival, bile resistance and acid production in skim milk culture were determined.

**Results:**

Equine whey proteins were present in airag samples more than caseins. The airag samples contained 0.10–3.36 % lactose, 1.44–2.33 % ethyl alcohol, 1.08–1.62 % lactic acid and 0.12–0.22 % acetic acid. *Lactobacillus* (*L.*) *helveticus* were major lactic acid bacteria consisting of 9 isolates among total 18 isolates of lactic acid bacteria. *L. helveticus* survived strongly in PBS, pH 3.0 but did not grow in MRS broth containing 0.1 % oxgall. A couple of *L. helveticus* isolates lowered pH of skim milk culture to less than 4.0 and produced acid up to more than 1.0 %.

**Conclusion:**

Highly variable biochemical compositions of the airag samples indicated inconsistent quality due to natural fermentation. Airag with low lactose content should be favorable for nutrition, considering that mares’ milk with high lactose content has strong laxative effect. The isolates of *L. helveticus* which produced acid actively in skim milk culture might have a major role in production of airag.

## Background

Airag, also called either koumiss or chigee, is a popular beverage traditionally produced in central Asian regions including Kazakhstan, Krygyzstan, some central regions of Russia and Xinjiang, Inner Mongolia and Qinghai of China as well as Mongolia. It is a mildly alcoholic, sour-tasting fermented drink made from unpasteurized fresh mares’ milk. Old airag made in the previous year or freshly prepared airag is mixed with fresh mares’ milk at the rate of 20 % [1]. The mixture is kept in a suitable bag which is made of animal hide. During fermentation, regular beating and storage temperature at 20–30 °C are required in order to produce acid, ethanol and flavors. The fermentation results in up to 2 % ethyl alcohol content and low pH less than 4 [2].

The major microorganisms in airag have been shown to be lactobacilli and yeasts in previous studies [3–6]. Lactobacilli convert lactose into lactic acid to acidify mares’ milk to provide refreshing sour taste. Yeasts ferment the sugar into ethyl alcohol and carbon dioxide to produce carbonated mildly alcoholic drink. The mixed culture of lactobacilli and yeasts in airag seems to enhance cell growth and fermentation better than single culture [7]. The ability of *Kluyveromyces marxianus* to utilize lactose effects its dominant growth in milk [3, 5]. The major *Lactobacillus* species isolated from airag of Mongolia and China is *Lactobacillus helveticus*, *Lactobacillus plantarum*, and *Lactobacillus casei* [4, 8–10]. The proportions of the *Lactobacillus* species in airag seem to be affected by recipes to prepare starter cultures for airag production and by geographical location [5, 11].

For long centuries, airag has been recognized as a wholesome beverage which influences alimentary canal, circulation system and immune system. Airag is claimed to belong to functional foods that provide health benefits [12], which result from high contents of polyunsaturated fatty acids in mares’ milk and probiotic microorganisms as well as basic nutrients, such as calcium and protein [13–15]. However, mares’ milk has been recognized as a strong laxative because of high content of lactose which cannot be digested and absorbed in the small intestine of most Asians in adult age. Since lactose can be decomposed into lactic acid and ethanol during fermentation, airag may not cause diarrhea and abdominal cramp, which are frequent symptoms of lactose intolerance.

The objectives of this study were to investigate microbiological and biochemical characteristics of airag samples collected in Ulaanbaatar, Mongolia and to determine physiological property and growth in skim milk of isolated lactic acid bacteria which might play major roles in producing acid in airag.

## Methods

### Sample collection

The airag samples were collected from Ulaanbaatar, Mongolia in 2013 and 2014. The samples were put into sterile polyethylene tubes and kept cold in an ice box during transportation by air to Laboratory of Food Biotechnology in Sangji University. Microbiological analysis of the samples stored at 4 °C was performed within a week of collecting them. The frozen samples were used for SDS-PAGE and HPLC.

### Enumeration and isolation of microorganisms

BCP plate count agar (Eiken Chemical, Japan) supplemented with 0.01 % cycloheximide and yeast malt extract agar supplemented with 0.01 % chloramphenicol [5] were used to enumerate lactic acid bacteria and yeasts, respectively. Enumeration was performed by spreading 0.1 mL of samples diluted with 0.1 % peptone on the agar plate. The plates for counting lactic acid bacteria and yeasts were incubated anaerobically at 32 °C for 3 days and aerobically at 25 °C for 4 days, respectively.

The diluted airag samples were spread on M17 agar (Difco, USA) which was supplemented with lactose and MRS agar (Difco, USA) which was acidified to pH 5.2. The M17 agar and MRS agar were then incubated anaerobically at 32 °C for 3 days to isolate enterococci and lactobacilli, respectively from the airag samples [16, 17]. The isolated colonies were scraped from the agar plates and suspended in skim milk. Equal volume of 20 % glycerol and skim milk was added to the suspension, which was then stored at -80 °C.

### Identification of lactic acid bacteria

Lactic acid bacteria were identified by sequencing 16 s rRNA gene and then by using BLAST search program (National Center of Biotechnology Information, USA). The sequencing experiments were provided by Solgent Co., Ltd, in Korea. The procedure was described briefly as follows: Genomic DNA was extracted from colonies on the agar plates by using purification beads (Solgent, Korea). The partial sequence from the forward primer 27 F was determined by using Bigdye terminator cycle sequencing kit (Perkin-Elmer, USA) and ABI Prism 3730x1 DNA Analyzer (Perkin-Elmer, USA).

### Phylogenetic relationship between lactobacilli

The partial sequences of 16S RNA genes of lactobacilli were modified by removing sequences at 5′-end and 3′-end to obtain a 909 bp-long sequence with 5′-ggggcccgcacaagcggtgg-3′ at 3′-end. The resulting sequences of the isolates were subjected to multiple sequence alignment programs of multiple alignment using fast fourier transform(MAFFT) which were provided by Kyoto University Bioinformatics Center(KUBC) and European Molecular Biology Laboratory(EMBL). A diagram of rooted phylogenetic tree with branch length and percent identities between lactobacilli were obtained from the programs provided by KUBC and EMBL, respectively.

### Physiological characteristics of lactic acid bacteria

Physiological characteristics were examined following the methods described by Harrigan and McCane [18]. Studies to determine ability of enterococci to grow at 10 °C, at 45 °C, at pH 9.6 and in the presence of 6.5 % sodium chloride and to survive heating at 60 °C for 30 min were performed using yeast glucose lemco broth. Production of ammonia from arginine was determined in the arginine broth. Ability of lactic acid bacteria to ferment carbohydrates was determined using MRS fermentation broth (Scharlau, Spain) which was supplemented with 2 % filter-sterilized carbohydrates.

### Acid survival of lactic acid bacteria

Two hundred microliters of cultures in MRS broth which had been incubated at 37 °C for 24 h were suspended into 20 mL of phosphate buffered saline, pH 7.2 (PBS, pH 7.2) and PBS, pH 3.0 which was acidified by adding HCl. After incubating at 37 °C for 3 h, lactic acid bacteria in the suspension were enumerated by spreading diluted suspension on BCP plate count agar which was incubated at 37 °C for 2 days. The percent of acid survival was calculated by dividing the bacterial number of PBS, pH 3.0 with that of PBS, pH 7.2 and then by multiplying with 100 % as described before [6].

### Bile resistance of lactic acid bacteria

Two hundred microliters of the same cultures used in the study of acid survival were suspended into 20 mL of MRS broth without addition (MRS-control) and MRS broth containing 1 % oxgall (MRS-bile). The inoculated broth was incubated at 37 °C for 24 h. The absorbance of the culture was measured at 600 nm. The percent of bile resistance was calculated by dividing absorbance of culture in MRS-bile with that of culture in MRS-control and then by multiplying 100 % as described before [6].

### Preparation of frozen concentrated culture

MRS broth containing lactose instead of glucose was prepared to produce frozen concentrated cultures of lactic acid bacteria. Lactic acid bacteria were cultured in 200 mL of the above broth at 37 °C for 24 h and centrifuged at 200xg for 30 min. The cell pellets were suspended into the equal mixture of skim milk and 20 % glycerol to make about 8 mL of cell suspension. Aliquots of the cell suspension were dispensed into tubes and frozen in the freezer at -80 °C.

### Fermentation of skim milk culture

The frozen concentrated cultures were used as inoculants for skim milk cultures of lactic acid bacteria isolated from the airag samples. Aliquots of the frozen concentrated cultures which had been thawed at room temperature immediately before experiments was added to make bacterial suspensions of 7 logCFU/mL in 200 mL of skim milk. The inoculated skim milk cultures were incubated at 25 °C, 30 °C and 37 °C for 4 days. Samples were taken to determine pH and titratable acidity and to enumerate lactic acid bacteria after 0, 1, 2, 3 and 4 days.

Skim milk cultures of *L. helveticus* isolates which were activated by incubating serially twice at 35 °C for 1 day were used as inoculants. Two milliliters of the skim milk cultures were suspended into 200 mL of skim milk. The inoculated skim milk cultures were incubated at 35 °C for 3 days. Samples were taken to determine pH of the cultures after 0, 1, 2 and 3 days.

### Chemical analysis

Titratable acidity was determined by following the method described by Bradely et al. [19]. The contents of lactose, lactic acid, acetic acid and ethyl alcohol were determined by using high performance liquid chromatography (HPLC). In order to make samples for HPLC analysis, the airag samples were acidified to pH 2.0–2.3 by adding 1 M sulfuric acid and centrifuged to 1,000xg for 30 min. The supernatants were passed through a column containing 0.2 g of Chelex 100 resin (Bio-Rad, USA) and filtered through a membrane filter with pore size of 0.45 μm. The filtrates were used as samples for the HPLC analysis. The HPLC system (Varian, USA) consisted of a solvent delivery system (9012Q), a refractive index detector (Star 9040), and a column oven (101). The temperature of Rezex ROA column with the size of 150x7.7 mm (Phenomenex, USA) was 60 °C. The eluant was 4 mM sulfuric acid and the flow rate was 0.6 mL/min.

### Sodium dodecyl sulfate-polyacrylamide gel electrophoresis (SDS-PAGE)

SDS-PAGE of airag sample was performed following the procedure described by Laemmli [20]. The airag samples were adjusted to pH 7 by adding 5 M NaOH and dialyzed in a cellulose membrane tubing (Sigma Aldrich, USA). Equal amounts of Laemmli’s sample buffer (2x) containing 5 % 2-mercaptoethanol were added to the samples, which were then heated at 95 °C for 5 min and then subjected to the electrophoresis. Separating gel of 14 % acrylamide was used for electrophoresis. Molecular weight standards (Bio-Rad, USA) used for SDS-PAGE were phosphorylase b (97,400), serum albumin (66,200), ovalbumin (45,000), carbonic anhydrase (31,000), trypsin inhibitor (21,500) and lysozyme (14,400). The unit of molecular weight number in parenthesis is dalton.

## Results and discussion

### Biochemical and microbiological characteristics of airag

**Fig. 1:**
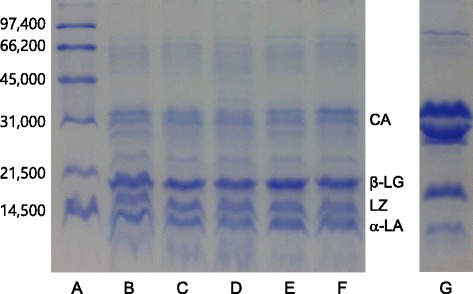
SDS-PAGE of airag samples collected in Ulaanbaatar, Mongolia. A: Molecular weight standards, B-F: the airag samples 1–5, G: cows’ skim milk, CA: caseins, β-LG: lactoglobulin, LZ: lysozyme, α-LA: α-lactalbumin, The numbers at left indicate molecular weights in dalton of the standards described in materials and methods

The polypeptide compositions of five airag samples were investigated by using SDS-PAGE (Fig. 1), which showed that three polypeptides with molecular weights near 30,000 daltons shown in lane B, C, D, E and F were equine caseins [21], which had similar molecular weights of bovine caseins shown in lane G. Equine whey proteins consisting of β-lactoglobulin, lysozyme and α-lactalbumin with the molecular weights of 18,800, 15,500 and 14,400 daltons, respectively, [22] were shown as major proteins in the five airag samples in the lane B, C, D, E and F. Bovine β-lactoglobulin and α-lactalbumin with the molecular weight of 18,300 and 14,400 daltons were shown in lane G, but bovine lysozyme which exists in trace amount in cows’ milk [13] could not be detected.

Equine whey proteins seemed to be major proteins in the airag samples in this study. Malacarne et al. [13] reported that the proportions of whey protein to crude protein in mares’ milk and cows’ milk were 38.79 and 17.54 %, respectively. Uniacke-Lowe et al. [23] showed that casein to whey ratios of mare milk and cow milk were 1.1 and 4.7, respectively. However, SDS-PAGE in Fig. 1 showed that the staining intensities of whey proteins in airag was stronger than those of caseins. This result suggested that equine caseins might be partially hydrolyzed during fermentation of mare’s milk. It was shown that up to 10 % of equine milk proteins were hydrolyzed after 96 h during fermentation of mares’ milk [23]. Egito et al. [21] reported that equine α- and β-casein were susceptible to cleavage by aspartate proteases such as chymosin.

The counts of lactic acid bacteria and yeasts in the airag samples were enumerated by using BCP plate counts agar supplemented with 0.01 % cycloheximide and yeast malt extract agar supplemented with 0.01 % chloramphenicol, respectively. Table 1 showed that the counts of lactic acid bacteria and yeasts and pH of the airag samples ranged 8.07–8.76 logCFU/mL, 6.43–7.87 logCFU/mL and 3.53–3.89, respectively. The contents of lactose, lactic acid, acetic acid and ethyl alcohol were variable among the airag samples and ranged 0.10–3.36 %, 1.08–1.62 %, 0.12–0.22 % and 1.44–2.33 %, respectively. The airag sample 1 showed the highest numbers of lactic acid bacteria and yeasts and the highest contents of lactic acid and ethyl alcohol of all the airag samples. The airag samples 3 and 4 contained 0.1 % lactose which indicated that most of lactose was spent. The airag sample 5 contained the highest amount of lactose and the lowest amount of ethyl alcohol of all the samples, which suggested deficient alcohol fermentation. Overall, the results showed that the compositions were highly variable between the airag samples suggesting inconsistent qualities due to natural fermentation.

**Table 1:** Microbiological and biochemical compositions of airag samples collected in Ulaanbaatar, Mongolia

No.	Lactic acid bacteria	Yeasts	pH	Lactose	Lactic acid	Acetic Acid	Ethyl alcohol
	logCFU/mL			%			
1	8.76	7.87	3.53	0.78	1.62	0.12	2.57
2	8.04	7.41	3.89	1.56	1.14	0.22	2.38
3	8.54	7.66	3.71	0.10	1.36	0.15	2.04
4	8.23	6.43	3.66	0.11	1.28	0.08	2.15
5	8.07	7.61	3.88	3.36	1.08	0.16	1.44

Sun et al. [4] reported that the counts of lactic acid bacteria and yeasts of the airag samples collected in Ulaanbaatar region of Mongolia ranged from 6.88 to 7.38 logCFU/g and 5.49 to 6.11 logCFU/g, respectively. pH ranged 3.7 to 4.1. Watanabe et al. [5] showed that 22 airag samples collected from Steppe, Forest-steppe, Gobi and Desert in Mongolia contained populations of lactic acid bacteria and yeasts at 7.78 and 7.41 logCFU/mL, respectively. Mu et al. [3] reported that 96 koumiss samples of China harbored yeast populations at 5–7 logCFU/mL. The microbiological characteristics of the airag samples in this study showed that the yeast counts were similar with those reported by the previous studies [3–5], but the lactic acid bacteria counts were higher than those reported by Sun et al. [4] and Watanabe et al [5]. The pH of the airag samples were lower than those reported by Sun et al. [4]. Thus, it seemed that the airag samples in this study underwent extended growth of lactic acid bacteria and strong production of lactic acid.

Table 1 showed that most of the airag samples contained low amounts of lactose. Thus, intake of airag shall not cause symptoms of lactose intolerance, even though mares’ milk with 6.4 % lactose [13] has strong laxative effects. Mongolian tradition of drinking airag as a staple food during summer seems to be an appropriate way to take in essential nutrients, such as calcium, vitamin, protein and essential fatty acid rich in mares’ milk [13–15] and probiotics present in airag [6, 9].

### Identification of lactic acid bacteria isolated from airag

Lactobacilli and enterococii were isolated by inoculating MRS agar acidified to pH 5.2 and M17 agar containing lactose, respectably, with the airag samples diluted with 0.1 % peptone and then by incubating anaerobically at 32 °C for 3 days. Eighteen isolates were identified based on 99–100 % of percent identity which was obtained by analyzing partial sequences of 16S rRNA gene on BLAST tool. Nine isolates of *Lactobacillus* (*L.*) *helveticus* SJU1-9, two isolates of *L. kefiranofaciens* SJU10-11, two isolates of *L. kefiri* SJU12-13, and an isolate of *L. diolivorans* SJU14, two isolates of *Enterococcus* (*E.*) *faecalis* SJU 15–16, an isolate of *E. faecium* SJU17 and an isolate *E. durans* SJU18 were identified. GenBank accession numbers of the partial sequences of 16S rRNA gene of the isolates, SJU1-18, are KT368983-KT369000, respectively.

Previous studies also showed that all the airag samples collected in various regions of Mongolia contained *L. helveticu*s as predominant lactic acid bacterial species [4, 5, 9, 10]. *L. helveticus* (19 isolates), *L. plantarum* (6 isolates) and *L. casei* (1 isolate) from five airag samples collected at households in Ulaanbaartar, Mongolia were identified by Sun et al. [4]. The major lactic acid bacteria of airag samples collected from three nomadic families in Donto-Govi prefecture in Mongolia consisted of *L. helveticus* and *L. kefiri* [10]. Watanabe et al. [5] reported that *L. helveticus*(21/22) and *L. kefiranofaciens* (14/22) were major lactic acid bacteria isolated from 22 airag samples in Mongolia. Takeda et al. [9] isolated 67 lactic acid bacteria from 7 airag samples collected in Ulaanbaatar, Mongolia. *L. helveticus*, *L. delbrueckii* ssp. *lactis*, and *L. fermentum* were dominant species at the levels of 46.2 %, 22.4 %, and 11.9 %, respectively.

However, An et al. [24] reported that *L. plantarum*, *L. pentosus* and *Lactococcus lactis* ssp. *cremoris* were the major lactic acid bacteria in chigee collected in Inner Mongolia, China at the rate of 48, 33 and 19 %, respectively. Wu et al. [6] reported that dominant lactobacilli species in koumiss sampled in Inner Mongolia, China were *L. casei*, *L. helveticus* and *L. plantarum* at the levels of 35, 21, and 17 %, respectively. Sun et al [8] reported that *L. helveticus*, *L. casei*, and *L. plantarum* appear to be dominant species in koumiss collected in Xinjiang, Inner Mongolia and Qinghai, China, respectively. These studies showed that the main lactobacilli in the koumiss samples collected in China were diverse and different from those in Mongolia.

There are many types of fermented milk including yogurt which is manufactured industrially in many countries by using defined cultures containing *Streptococcus thermophilus* and *L. delbrueckii* ssp. *bulgaricus* [25]. Kefir is a drink which is prepared from cows’ milk by using kefir grains as a starter culture which consists of *Lactococcus lactis*, *L. acidophilus*, *L. kefir*, *L. kefiranofaciens*, *L. casei*, *Candida kefyr*, *Kluyveromyces marxianus* and *Saccharomyces cerevisiae.* Kefir contains 0.5–1.0 % ethyl alcohol, whereas the airag samples in this study contained 1.44–2.33 %. Koumiss is also manufactured commercially by using various lactic acid bacteria and *Kluyveromyces marxianus* as starter [25]. Three types of koumiss exist, so-called ‘strong’,’moderate’ and ‘light’ koumiss depending on the lactic acid contents [26]. ‘Strong ‘koumiss is generated by lactic acid bacteria, such as *L. delbrueckii* ssp. *bulgaricus*, which acidify the milk to pH 3.6–3.3.’Moderate’ koumiss contains lactic acid bacteria, such as *L. acidophilus*, *L. plantarum* and *L. casei* that lower the pH to 4.5–3.9. ‘Light’ koumiss is a slightly acidified product with pH 4.5–5.0 and harbors *Lactococcus lactis* and *Streptococcus thermophiles.* The airag samples in this study could be classified as ‘moderately strong’ koumiss.

### Phylogenetic relationship analysis

**Fig. 2:**
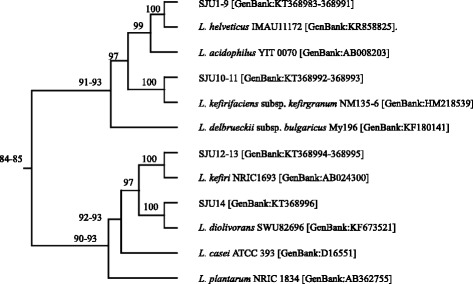
Rooted phylogenetic tree with branch length of lactobacilli isolated from airag samples and lactobacilli strains in NCBI database. The number in the diagram is percent identity. The word in parenthesis is accession number of nucleotide sequence in NCBI database

Phylogenetic relationships among lactobacilli including the isolates from the airag samples and other lactobacilli used as commercial starter cultures for yogurt and probiotics were determined by comparing the partial nucleotide sequences of 16S rRNA genes of 909 bp in the multiple sequence alignment program of MAFFT. The MAFFT analysis showed that the sequences adjacent to the 5′-end of the partial sequences of 16S rRNA genes were highly variable among the lactobacilli, but the sequences adjacent to the 3′-end were similar each other (data not shown). Figure 2 showed a diagram of rooted phylogenetic tree with branch length and percent identity numbers which were derived from the MAFFT analysis. The diagram showed that *L. helveticus* and *L. kefiranofaciens* were closely related with each other at 99 % identity and moderately associated at 97 % identity with *L. acidophilus. L. diolivorans* and *L. kefiri* showed moderate relationship at 97 % identity. The phylogenetic relationship of the lactobacilli in Fig. 2 corresponded to the classification proposed by Hammes and Hertel [27]. *L. helveticus*, *L. kefiranofaciens* and *L. acidophilus* belongs to Group A lactobacilli which is obligately homofermentative. *L. diolivorans* and *L. kefiri* are among Group B lactobacilli which is facultatively heterofermentative.

*L. acidophilus* is one of the important probiotics used to produce therapeutic fermented milks [28]. Many studies showed that *L. helveticus* and *L. kefiranfaciens* were also good candidates to become probiotics. The species of *L. helveticus* are highly proteolytic lactic acid bacteria which produce peptides which possess inhibitory activity on the angiotensin converting enzyme (ACE) [29, 30] and are traditionally used in the manufacture of Swiss-type cheeses and long ripened Italian cheeses [31]. *L. kefiranfaciens* was reported to produce polysaccharide, e.g. kefiran, which exerts hypocholesterolemic and hypotensive effects [32, 33].

### Physiological characteristics of lactic acid bacteria

Physiological characteristics of *Lactobacillus* isolates (Table 2) and *Enterococcus* isolates (Table 3) were investigated. *L. helveticus* SJU1-9 were classified into A and B groups based on carbohydrate fermentation. Seven isolates (SJU1-7) in the A group fermented fructose, galactose, glucose, lactose and mannose and two isolates (SJU8-9) in the B group fermented additional carbohydrates including maltose, melibiose, salicin, sucrose, and trehalose. *L. kefiranfaciens* SJU10-11 utilized 16 carbohydrates except arabinose, raffinose, rhamnose and xylose. *L. kefiri* SJU 12–13 produced acid from fructose, galactose, lactose, maltose and melibiose, but not from glucose. In fact, *L. kefiri* SJU 12–13 grew poorly in MRS broth but showed heavy growth in MRS broth containing lactose instead of glucose in this study (results not shown). Table 3 showed that all the *Enterococcus* isolates (SJU15-18) produced ammonia from arginine. *E. faecalis* SJU15-16 and *E. faecium* SJU17 grew at 45 °C and after heat treatment at 60 °C, but *E. durans* SJU18 did not. *E. faecalis* SJU15-16 produced acid from 15 carbohydrates except arabinose, melibiose, raffinose, rhamnose and xylose. *E. faecium* SJU17 and *E. durans* SJU18 produced acid from relatively fewer carbohydrates than *E. faecalis* SJU15-16.

**Table 2:** Carbohydrate fermentation characteristics of lactobacilli isolated from the airag samples

Identification	*L. helveticus*		*L. kefiranofaciens*	*L. kefiri*	*L. diolivorans*
Groups	A	B			
Isolates	SJU1-7	SJU8-9	SJU10-11	SJU12-13	SJU14
Arabinose	-	-	-	(1)^*^	+
Cellobiose	-	(1)^*^	+	-	
Fructose	+	+	+	+	+
Galactose	+	+	+	+	+
Gluconate	-	-	+	-	+
Glucose	+	+	+	-	+
Lactose	+	+	+	+	+
Maltose	-	+	+	+	+
Mannitol	-	-	+	-	
Mannose	+	+	+	-	
Melezitose		-	+	-	
Melibiose	-	+	+	+	+
Raffinose	-	-	-	-	-
Rhamnose	-	-	-	-	-
Ribose	-	-	+	(1)^*^	-
Salicin	-	+	+	-	+
Sorbitol	-	-	+	-	
Sucrose	-	+	+	-	+
Trehalose	-	+	+	-	+
Xylose	-	-	-	-	-

*The number in parenthesis is the number of positive isolate

**Table 3:** Physiological characteristics of enterococci isolated from the airag samples

Identification		*E. faecalis*	*E. faecium*	*E. durans*
Isolates		SJU15-16	SJU17	SJU18
Growth at	10 °C	+	+	+
	45 °C	+	+	-
	pH 9.6	+	+	+
	6.5 % NaCl	+	+	+
Heat resistance at 60 °C		+	+	-
Ammonia from arginine		+	+	+
Fermentation of				
	Arabinose	-	-	-
	Cellobiose	+	+	+
	Fructose	+	+	+
	Galactose	+	+	+
	Gluconate	+	-	-
	Glucose	+	+	+
	Lactose	+	+	+
	Maltose	+	+	+
	Mannitol	+	-	-
	Mannose	+	+	+
	Melezitose	+	-	-
	Melibiose	-	+	-
	Raffinose	-	-	-
	Rhamnose	-	-	-
	Ribose	+	+	+
	Salicin	+	+	+
	Sorbitol	+	-	-
	Sucrose	+	-	-
	Trehalose	+	+	-
	Xylose	-	-	-

The physiological characteristics of the isolates of lactobacilli and enterococci described in Table 2 and Table 3, respectively were comparable to those described previously [18, 27, 34]. Sun et al. [8] reported that more than eighty percent of 114 *L. helveticus* isolates from koumiss in China produced acid from fructose, galactose, lactose, maltose, mannose and sucrose. Two *L. kefiranofaciens* isolates produced acid from amygdalin, cellobiose, dextrin, fructose, galactose, inositol, inulin, lactose, maltose, mannitol, mannose, melezitose, sarlicin, sorbitol, sucrose and trehalose.

### Acid survival and bile resistance

Acid survival in PBS, pH 3.0 and bile resistance in MRS broth with 0.1 % oxgall (MRS-bile) of isolated lactic acid bacteria were investigated (Table 4). *L. helveticus* SJU1, SJU5 and SJU7 and *L. kefiranofaciens* SJU11 showed up to 100 % acid survival. *L. helveticus* SJU1, SJU5, SJU7 and SJU9 and *L. kefiranofaciens* SJU11 did not grow in MRS-bile. *E, faecalis* SJU15 and SJU16 and *E. durans* SJU18 grew in MRS-bile strongly. *E. faecalis* SJU15 with moderately strong acid survival and with strong bile resistance seemed to be a good candidate for probiotics in terms of ability to pass through stomach and to multiply in the intestine. Further researches are necessary to isolate appropriate *Lactobacillus* probiotics from airag.

**Table 4:** Acid survival and bile resistance of lactic acid bacteria isolated from the airag samples

Identification	Isolates	Acid survival^a^	Bile resistance^a^
		%	%
*L. helveticus*	SJU1	115 ± 11	0
	SJU5	103 ± 21	0
	SJU7	98 ± 27	4 ± 0
	SJU9	57 ± 7	0
*L. kefiranofaciens*	SJU10	53 ± 8	2 ± 0
	SJU11	131 ± 56	4 ± 0
*L. kefiri*	SJU12	50 ± 19	11 ± 2
	SJU13	72 ± 35	26 ± 0
*L. diolivorans*	SJU14	44 ± 29	49 ± 7
*E. faecalis*	SJU15	75 ± 7	101 ± 13
	SJU16	31 ± 5	129 ± 40
*E. faecium*	SJU17	51 ± 5	30 ± 0
*E. durans*	SJU18	57 ± 21	119 ± 1

^a^mean ± standard deviation

Many studies have isolated probiotic lactic acid bacteria from airag in Mongolia and koumiss in China based on tolerance in gastrointestinal environment, antimicrobial property and adhesion to cell such as Caco-2 cell [6, 9, 35, 36]. Wu et al [6] isolated potential probiotics through the preliminary tests including resistance to high acid and abilities to grow in MRS with bile salts. *L. casei* Zhang survived strongly in PBS, pH 3.0 and could grow in MRS broth with 1.0 % bile. Rong et al. [35] isolated *L. helveticus* NS8 from koumiss in Inner Mongolia, China as a candidate for probiotics which showed higher tolerance to low pH (70 % survival rate at pH 2) and toxic bile salts (65 % survival rate in 0.3 % bile). Takeda et al. [9] reported that *L. plantarum* 05 AM23 isolated from airag samples in Ulaanbaatar, Mongolia showed presumable probiotic characteristics of strong bile acid tolerance, high viability at low pH and strong adhesion on Caco-2 cell. Bilige et al. [36] screened *L. helveticus* isolates from home-made airag in Mongolia and identified *L. helveticus* MG2-1 which is most effective in tolerance to artificial gastrointestinal juice, sodium taurocholate deconjugation, cholesterol removal and adhesion to Caco-2 cell.

### Acid production in skim milk cultures

**Fig. 3:**
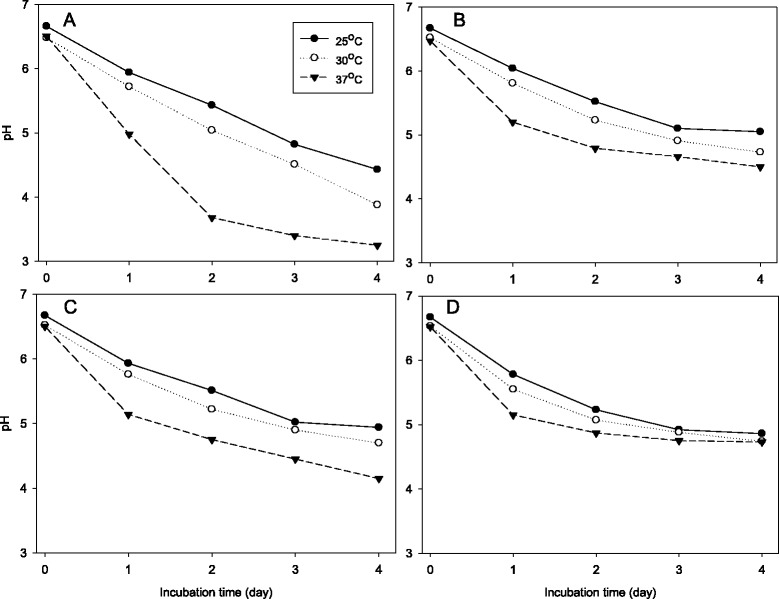
Change of pH of skim milk cultures which were inoculated with frozen concentrated cultures of lactic acid bacteria at the level of 7 logCFU/mL and incubated at 25, 30 and 37 °C. **a**: *L. helveticus* SJU5, **b**: *L. kefiranofaciens* SJU10, **c**: *L. kefiri* SJU12, **d**: *E. faecalis* SJU16

**Fig. 4:**
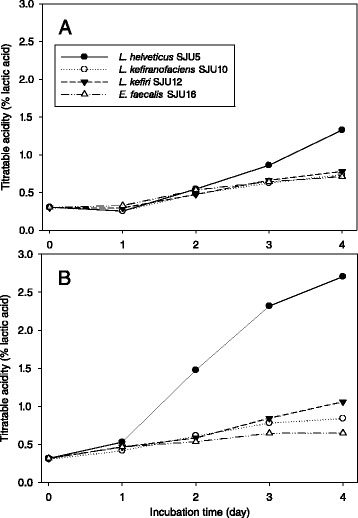
Change of titratable acidity of skim milk cultures which were inoculated with frozen concentrated cultures of lactic acid bacteria at 7 logCFU/mL and incubated at 30 °C (**a**) and 37 °C (**b**)

Changes of pH (Fig. 3), titratable acidity (Fig. 4) and bacterial number (Table 5) of skim milk cultures of *L. helvetcius* SJU5, *L. kefiranofaciens* SJU10, *L. kefiri* SJU12 and *E. faecalis* SJU16 were investigated. The frozen concentrated cultures were used to inoculate skim milk at the level of 7 logCFU/mL. Figs. 3 and 4 showed that all of the lactic acid bacteria produced acid and thus lowered pH of the skim milk culture faster at 37 °C than at the lower temperatures of 30 and 25 °C. The skim milk cultures of *L. helveticus* SJU5 reached pH 3.25 and 2.71 % titratable acidity for 4 days at 37 °C. Neither *L. kefiranofaciens* SJU10 nor *E. faecalis* SJU12 reached pH 4.5 for 4 days at 37 °C. Table 5 confirmed that all the numbers of lactic acid bacteria just after inoculation were close to 7 log CFU/per mL. These results showed that *L. helveticus* SJU5 produced lactic acid more than the other three lactic acid bacteria.

**Table 5:** Growth of lactic acid bacteria during fermentation of skim milk cultures which were inoculated at the level of 7 logCFU/mL and incubated at 25 °C

Incubation time (day)	*L. helveticus* SJU5 (logCFU/mL)	*L. kefiranofaciens* SJU10	*L. kefiri* SJU12	*E. faecalis* SJU16
0	6.87	6.97	6.89	6.87
2	8.80	8.84	8.73	9.04
4	9.07	9.79	9.17	10.05

**Fig. 5:**
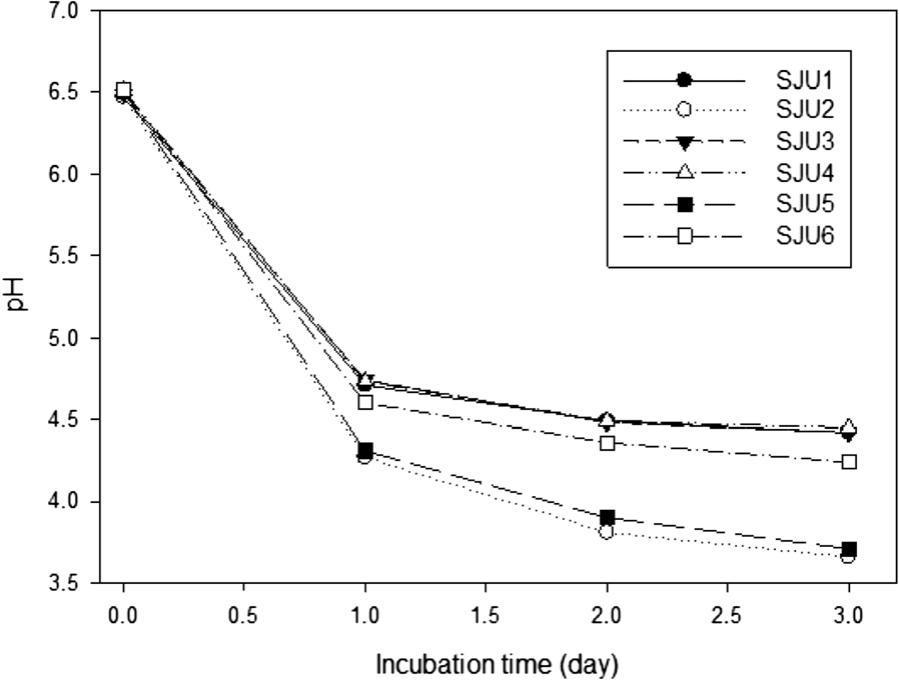
Change of pH of skim milk cultures of *L. helveticus* isolates SJU1-6 which were inoculated with 1 % skim milk cultures and incubated at 35 °C

Figure 5 showed changes of pH of skim milk culture at 35 °C of *L. helveticus* CJU1-6. Two milliliters of activated skim milk cultures were used to inoculate 200 mL of skim milk which was subsequently incubated to determine pH changes. *L. helveticus* CJU2 and CJU5 decreased pH to 3.76 and 3.71, respectively, for 2 days. These results suggested that *L. helveticus* CJU2 and CJU5 should have played major roles in producing lactic acid in the airag samples collected in Mongolia in this study.

Wulijideligen et al. [7] reported that *L. helveticus* I30B4 fermented skim milk at 30 °C to increase titrable acidity to 2.57 % for 7 days, However, *L. plantarum* 440 M6, *L. casei* 6B4084 and *L. paracsei* 6B3073 increased titratable acidity to 1.41 %, 1.25 % and 1.26 %, respectively. The titratatable acidities of skim milk cultures of *L. reuteri* 940B3 and lactococci did not reach 1.0 %. The results in this study also showed that *L. helveticus* was more effective in producing lactic acid during fermentation of skim milk than the other lactic acid bacteria.

*L. helveticus*, which has been most frequently isolated from airag in Mongolia, is also used traditionally used in cheese-making in Swiss and Italy and is recovered from the natural lactic acid starter cultures used for the production of Italian cheese. It is also gaining potential as health-promoting probiotic in dietary supplement [31]. *L. helveticus* strains isolated from cheese showed characteristics of rapid lysis and high proteolytic activity [37]. The highly efficient proteolytic system of *L. helveticus* enhances texture and flavor during cheese aging and produces bioactive peptides from milk proteins in fermented milk beverage which may provide beneficial effects on blood pressure, immune system, calcium absorption and anti-virulent effects against pathogens [38]. The strong acid production capacity and proteolytic activity of *L. helveticus*, the major lactic acid bacteria in airag, should enable Mongolian to enjoy flavorful beverage which provide therapeutic benefits as well as essential nutrients.

## Conclusions

The compositions, particularly lactose contents, of the airag samples collected in Ulaanbaatar, Mongolia were variable, showing inconsistent quality due to natural fermentation. The low lactose content of airag may allow Mongolian to consume it in large quantity without suffering from lactose intolerance. Equine whey proteins rather than caseins were major proteins in the airag samples. pHs and titratable acidity of all the airag samples were less than 4.0 and more than 1 %, respectively, suggesting that airag was strongly sour-tasting. A couple of *L. helveticus* isolated from the airag samples produced lactic acid actively, which suggested that *L. helveticus* should have played a major role in fermentation of mares’ milk to produce airag.
